# An Approach to Detect Chronic Obstructive Pulmonary Disease Using UWB Radar-Based Temporal and Spectral Features

**DOI:** 10.3390/diagnostics13061096

**Published:** 2023-03-14

**Authors:** Hafeez-Ur-Rehman Siddiqui, Ali Raza, Adil Ali Saleem, Furqan Rustam, Isabel de la Torre Díez, Daniel Gavilanes Aray, Vivian Lipari, Imran Ashraf, Sandra Dudley

**Affiliations:** 1 Faculty of Computer Science and Information Technology, Khwaja Fareed University of Engineering and Information Technology, Rahim Yar Khan 64200, Pakistan; 2 School of Computer Science, University College Dublin, Dublin 4, D04 V1W8 Dublin, Ireland; 3 Department of Signal Theory, Communications and Telematics Engineering, Unviersity of Valladolid, Paseo de Belén, 15, 47011 Valladolid, Spain; 4 Research Group on Foods, Nutritional Biochemistry and Health, Universidad Europea del Atlántico, Isabel Torres 21, 39011 Santander, Spain; 5 Research Group on Foods, Nutritional Biochemistry and Health, Universidade Internacional do Cuanza, Cuito EN250, Bié, Angola; 6 Research Group on Foods, Nutritional Biochemistry and Health, Fundación Universitaria Internacional de Colombia, Bogotá 11001, Colombia; 7 Research Group on Foods, Nutritional Biochemistry and Health, Universidad Internacional Iberoamericana, Campeche 24560, Mexico; 8 Research Group on Foods, Nutritional Biochemistry and Health, Universidad Internacional Iberoamericana, Arecibo, PR 00613, USA; 9 Department of Information and Communication Engineering, Yeungnam University, Gyeongsan 38541, Republic of Korea; 10 School of Engineering, London South Bank University, 103 Borough Road, London SE1 0AA, UK

**Keywords:** chronic obstructive pulmonary disease, machine learning, ultra wideband radar, feature engineering

## Abstract

Chronic obstructive pulmonary disease (COPD) is a severe and chronic ailment that is currently ranked as the third most common cause of mortality across the globe. COPD patients often experience debilitating symptoms such as chronic coughing, shortness of breath, and fatigue. Sadly, the disease frequently goes undiagnosed until it is too late, leaving patients without the care they desperately need. So, COPD detection at an early stage is crucial to prevent further damage to the lungs and improve quality of life. Traditional COPD detection methods often rely on physical examinations and tests such as spirometry, chest radiography, blood gas tests, and genetic tests. However, these methods may not always be accurate or accessible. One of the key vital signs for detecting COPD is the patient’s respiration rate. However, it is crucial to consider a patient’s medical and demographic characteristics simultaneously for better detection results. To address this issue, this study aims to detect COPD patients using artificial intelligence techniques. To achieve this goal, a novel framework is proposed that utilizes ultra-wideband (UWB) radar-based temporal and spectral features to build machine learning and deep learning models. This new set of temporal and spectral features is extracted from respiration data collected non-invasively from 1.5 m distance using UWB radar. Different machine learning and deep learning models are trained and tested on the collected dataset. The findings are promising, with a high accuracy score of 100% for COPD detection. This means that the proposed framework could potentially save lives by identifying COPD patients at an early stage. The k-fold cross-validation technique and performance comparison with the state-of-the-art studies are applied to validate its performance, ensuring that the results are robust and reliable. The high accuracy score achieved in the study implies that the proposed framework has the potential for the efficient detection of COPD at an early stage.

## 1. Introduction

Chronic obstructive pulmonary disease is a life-threatening progressive pulmonary syndrome that causes breathlessness and may lead to death if not diagnosed and treated at early stages. COPD reduces the lung’s breathing capacity, creating respiration problems in patients. The primary element of COPD is emphysema and chronic bronchitis [1]. Emphysema is a pulmonary condition in which the lung tissues of patients are damaged. Chronic bronchitis is a bronchial condition caused by excessive coughing and mucus production in the respiratory tract. The most common symptoms of COPD are wheezing, shortness of breath and coughing. Coronary heart disease, weight loss, obesity, cognitive dysfunction, anorexia, and lung cancer indicate COPD [2]. According to a recent report by the world health organization (WHO), COPD is the third leading cause of death worldwide and has caused around 3.23 million deaths in 2019 alone [3]. The research shows that COPD has an extremely high mortality rate.

COPD is diagnosed by analyzing the patient’s history of exposure to pulmonary irritants such as smoking and family history. Currently, the doctor performs a physical examination to diagnose COPD in the hospital. The doctor uses classical methods such as a stethoscope to listen to a patient’s lung and heart sounds. In addition, several tests can also be performed for COPD detection including spirometry, chest radiography (X-ray), blood gas test, a computed tomography (CT) scan, and genetic tests. The spirometry examination is another best method for diagnosing COPD [4]. During the spirometry examination, the patient has to inhale to full lung capacity and then push the air out of the lungs as quickly as possible. The patient must maintain this examination until the lung volume is close to the residual volume. The efficiency of the spirometry examination raised several questions [5,6]. The improperly trained staff and inadequate testing are the reasons for low-quality spirometry results. By examining the classical method’s efficiency, advanced systems must be built to detect COPD in patients.

The ultra-wideband (UWB) radar is gaining wide attraction and is a highly used technology by the time domain and Xtreme spectrum companies [7,8]. The UWB radar has high data rates and low levels of transmission power. The UWB radar has non-intrusive and non-tackling capabilities to penetrate various obstacles or materials, which is an advantage over other classical methods [9,10]. Nowadays, many experiments are held in healthcare applications using wireless sensing systems based on UWB to identify vital signs related to different diseases [10,11]. The UWB radar radiates and absorbs little electricity compared to other instruments in biomedical applications.

Machine learning and deep learning are the subfields of artificial intelligence. Artificial intelligence-based techniques provide systems with the ability to automatically learn and improve from experience without being explicitly programmed to perform the task. In recent years, machine learning and deep learning have been applied to various bioinformatics problems such as drug discovery, protein structure prediction, disease detection, and many more. Machine learning and deep learning techniques can help us analyze large biomedical datasets to find new insights and develop new applications for bioinformatics research. Several COPD detection algorithms have been presented that utilize machine and deep learning methods. However, such methods are predominantly intrusive and lack the desired prediction accuracy. Situations such as pandemics require non-invasive methods that can detect COPD patients from a distance and can provide high accuracy. This study uses the temporal and spectral features data extracted from UWB radar-based biological signals to detect COPD paints. Machine learning and deep learning-based methods detect COPD patients from the temporal and spectral features data. The major contributions of this study are as follows:

- A new dataset is created in this study to detect COPD patients. The dataset is based on the UWB radar, which is used to collect data from confirmed patients and healthy people from a hospital.
- This study utilizes the temporal and spectral features from UWB radar biological signals data. The exploratory data analysis is applied to discover dataset patterns and correlations. The correlations analysis is conducted to select the dataset features with high correlation values that result in high performance.
- For experiments, four machine learning, and two deep learning model are employed for performance comparison. The decision tree (DT), logistic regression (LR), Gaussian Naive Bayes (GNB), and support vector machines (SVM) are the applied machine learning methods while long short-term memory (LSTM) and gated recurrent unit (GRU) are applied as deep learning models.
- Performance is also validated using k-fold cross-validation, as well as performance comparison with existing state-of-the-art studies.

The study is further divided into several sections. Section 2 analyzes the literature on COPD detection. The materials and methods of the proposed framework are examined in Section 3. The experimental results and discussions are presented in Section 4. In the end, Section 5 concludes the study findings for COPD detection.

## 2. Related Work

In view of the increasing number of deaths from COPD, several research works have been presented during the past years. For example, the goal of [12] is to examine whether or not UWB radar could be used as a non-invasive method for distinguishing between COPD patients and healthy individuals. Raw data are obtained from a distance of 1.5 m in a real-world setting (a hospital). The obtained raw data are then processed using signal extraction methods to obtain respiration data. The detection of COPD patients based just on the respiratory rate is insufficient. However, the performance is significantly improved by including other factors such as age, gender, and smoking history. Several machine learning classifiers are used to identify COPD cases including Naive Bayes (NB), SVM, random forest (RF), K nearest neighbor (KNN), Adaboost, and deep-learning models such as convolutional neural network (CNN), and LSTM. The findings of the experiments indicate that LSTM has the highest accuracy of 93%.

The study [13] determines the role of therapy to slow or stop disease development, especially severe COPD. Twelve channel recordings of lung function are examined for variable levels of COPD using the RespiratoryDatabase@TR. Forty-one patients’ right and left posterior (chest) and anterior (back) clinical auscultation sites are used to capture lung sounds. To isolate distinctive anomalies in lung sounds, a 3D second-order difference plot is used. Quantization based on the cuboid and octant is used to isolate signature anomalies on the chaos plot. In the classification phase, the deep extreme learning machine classifier (deep ELM), one of the most reliable and speedy deep learning algorithms, is used. Compared to the standard ELM autoencoder, the novel HessELM and LuELM autoencoder kernels are applied to deep ELM, resulting in improved generalization abilities and a quicker training time. The overall accuracy, weighted sensitivity, weighted specificity, and area under the curve (AUC) value of the proposed deep ELM model with LuELM autoencoder for classifying COPD severity are 94.31%, 94.28%, 98.76%, and 0.9659, respectively.

Electrocardiograph (ECG)-derived respiration (EDR) is used to differentiate between COPD patients and healthy individuals in [14]. The MP45 Biopac is used to record the heart rates and breath rates of 30 people during experiments. After examining the morphological pattern shifts in the respiration and EDR signals, three statistical characteristics are generated for each subject including area, time, and skewness ratio. Error computation and statistical analysis are used to establish how closely the EDR signal matches the original respiration signal. DT, linear discriminant analysis (LDA), SVM, and KNN classifiers are utilized for classification. An accuracy of 98.33% is found for respiration and EDR-derived features when using both DT and KNN.

The researchers in [15] employed a risk prediction strategy based on deep learning to detect COPD automatically by monitoring respiration rates. In order to distinguish between COPD and non-COPD, several feature combinations were employed using LR, DT, LDA, KNN, SVM, and quadratic discriminant classifiers. Spirometry readings and other parameters of respiration were used to identify the category. When using the two most important characteristics for interpreting lung sounds—median frequency and linear predictive parameters—the SVM classifier can attain a maximum classification accuracy of 83.6%. Using median frequency, linear predictive coefficient, and spirometry data, SVM and LR both obtained 99 percent accuracy.

An ECG- and EDR-based technique is proposed in [16] to detect subtle and obstructive respiratory disorders. The heartbeat data are collected using a Biopac system MP45. Each patient has an electrocardiogram recorded for 300 s at a sampling rate of 1000 hertz. Both the ECG and the EDR signal’s morphological variations are used to obtain temporal information, which is then used to identify distinctive features. The subjects are then categorized into normal, obstructive, and restrictive clusters utilizing numerous supervised classifiers. Evaluation of the classifier’s performance on 90 participants (both healthy and unwell) reveals that the SVM has a classification accuracy of more than 98%.

The study [17] discovers and compares the informative features of lung sounds using various signal processing techniques, as well as chooses the classification approach that gives the most accurate detection of bronchopulmonary system conditions. Power spectrum density (PSD) is estimated for respiratory signals using the Fast Fourier transform (FFT) technique. The spectrograms of the obtained signals are examined to derive the spectral features of the lung sounds. The average temporal dependences of the PSD at various frequencies are calculated. As spectrogram features, the sum of magnitude values of the power spectrum curve for each frequency band is used. The ratios of energies related to the detail levels of wavelet decomposition to the overall energy of the decomposed signal are employed as the parameters for wavelet analysis-based signal identification. As characteristics produced from mel-cepstral analysis, it is recommended to employ the logarithmic (mel) filterbank energies, averaged across time frames, depending on the channel index and time, as well as the mel frequency cepstrum based on cepstrum index. The best classification models for computerized illness screening are determined using supervised machine learning based on decision trees, discriminant analysis, SVM, LR, KNN, and ensemble learning. Using these feature sets, the accuracy of the various classifiers is calculated and compared. Based on the results, a combination of characteristics and classifiers with an identification accuracy of 93% for lung conditions is presented.

In the same manner, ref. [18] employs CNN to aid medical professionals by offering a comprehensive and rigorous analysis of the medical respiratory audio data for COPD identification. Librosa machine learning library features such as MFCC, Mel-Spectrogram, Chroma, Chroma (Constant-Q), and Chroma CENS are utilized for this purpose. Additionally, the proposed system could interpret the degree of the discovered ailment, such as mild, moderate, or severe. The findings of the research verify the effectiveness of the suggested deep learning method that achieved an accuracy score of 93%.

A CNN-based model is developed by [19] to diagnose COPD using the 3D lung airway tree. After extracting airway trees from CT scans, ventral, dorsal, and isometric snapshots of their 3D representations are generated. Using snapshots of each image, a deep CNN model is developed and then tuned using a Bayesian optimization approach in order to identify COPD. The ultimate forecast is determined by the majority vote of three opinions. The class-discriminatory localization maps have been created to graphically illustrate the CNNs’ judgments. The accuracy of the models trained with a single view (ventral, dorsal, and isometric) of colorful images are comparable (86.8%, 87.5%, and 86.7%), while the model after voting reaches an accuracy of 88.2%. Using gray and binary snapshots, the final voting model obtains an accuracy of 88.6% and 86.6%, respectively. Similarly, the study [20] proposes an integrated model for diagnosing COPD patients, based on the knowledge graph. First, a knowledge graph of COPD is developed in order to assess the link between feature subsets and identify knowledge about illnesses revealed by the data. Second, an algorithm for sorting features and an adaptive feature subset selection method, CMFS-, are proposed. CMFS- picks an ideal subset of features from the original high-dimensional collection. Finally, the DSA-SVM integrated model is used as a classifier for the diagnosis and prediction of COPD that achieved an accuracy of 95.1%.

The accurate analysis of respiratory tract fluids, such as saliva, can be a promising approach for identifying the severity of the disease and predicting its future exacerbations in a Point-of-Care (PoC) environment. However, it is important to take the demographic and medical parameters of patients into account to obtain accurate results. The study [21] applied machine learning techniques on saliva samples from COPD patients and healthy people, along with demographic information, for PoC recognition of the disease. As part of the Exasens joint research project, two sets of saliva samples were gathered from healthy controls (HC) and COPD patients. The samples consist of 160 HC and 79 COPD patient specimens and were collected at the BioMaterialBank Nord in Borstel, Germany between November 2016 and February 2018. A permittivity biosensor was used to analyze the dielectric properties of saliva samples. The XGBoost gradient boosting algorithm achieved a high classification accuracy and sensitivity of 91.25% and 100%, respectively, indicating its potential for COPD detection.

The study [22] compared several machine learning algorithms to identify early-stage COPD using multichannel lung sounds. The study analyzed multichannel lung sounds using statistical features of frequency modulations extracted using the Hilbert–Huang transform. The proposed deep learning model with Hilbert–Huang transform-based statistical features achieves high classification rates of 93.67%, 91%, and 96.33% for accuracy, sensitivity, and specificity, respectively. The analysis of multichannel lung sounds provides a standardized evaluation with high classification performance, and the 12-channel lung sound analysis provides the advantage of assessing entire lung obstructions. This study is the first to directly focus on lung sounds to differentiate between COPD and non-COPD patients, and its significance lies in its ability to provide a standardized assessment using advanced machine learning algorithms.

The authors analyzed the impact of different features for COPD detection in [23] with a focus on differentiating between early and advanced stages of the disease. The recursive feature elimination cross-validated (RFECV) method was utilized for feature selection, and expert doctors were consulted to recommend features among those selected using the RFECV method. Two sets of features were selected, and different machine learning algorithms were employed to compare their performance and feature importance. The RFECV method produced an accuracy of 96%, while feature reduction with doctor recommendation (FRDR) achieved an accuracy of 90%. Despite the slight difference in results, both sets of features exhibited promising outcomes.

The above-discussed studies report good results for COPD detection using various technologies and approaches. However, these studies have several limitations regarding the used technology or approach. Traditional approaches predominantly utilize physical examinations, in addition to several tests such as spirometry, chest radiography, blood test, and genetic tests. Yet, such tests are invasive, requiring close contact with the device and other people. In addition, the diagnosis is based on a doctor’s subjective evaluation and may be prone to error or misjudgments. Pandemic situations such as COVID-19, where physical contact is restricted, demand non-invasive technologies. In such scenarios, the proposed UWB-based approach is potentially important. Other than that, the reported accuracy of the discussed research works requires further improvement. The timely detection of COPD patients is another important aspect where UWB can be very effective. In addition, it has the capability to penetrate various obstacles or materials, which means that it can detect COPD patients through clothing and other obstructions. It also has low levels of transmission power and can radiate and absorb little electricity compared to other instruments in biomedical applications, which makes it safer for patients.

## 3. Study Methodology

**Figure 1:**
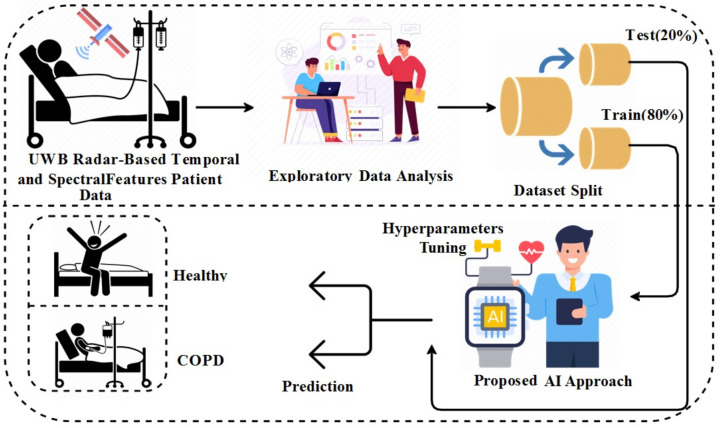
Work flow of the COPD patient detection.

Figure 1 shows the workflow diagram of the methodology used in this study. The newly created dataset based on the UWB radar is utilized in this study for experiments. The study makes use of temporal and spectral features for COPD detection. The exploratory data analysis is applied based on numerous insightful charts and graphs. The final dataset is then split into 80% to 20% for training and testing, respectively. The training portion is used for training the applied machine learning and deep learning models. The successfully trained models are then tested with the test data. The recursive process of hyperparameter tuning is applied to find each model’s best hyperparameters, resulting in high performance. All applied machine learning and deep learning models are optimized to obtain better results.

### 3.1. Temporal and Spectral Data Analysis

The temporal and spectral features-based dataset is created and utilized to conduct the study experiments [12]. The temporal and spectral features [24] are extracted from UWB radar-based biological signals. The features ‘energy entropy’, ‘short time energy’, ‘time zero crossing rate’, ‘spectral crest factor’, ‘time Rms’, ‘spectral kurtosis’, ‘spectral rolloff’, ‘spectral skewness’, ‘spectral flatness’, ‘spectral decrease’, ‘spectral centroid’, ‘spectral spread’, ‘spectral slope’, and ‘spectral flux’ are the extracted temporal and spectral features from the raw UWB signal. The descriptive analysis of the used study features are analyzed in Table 1.

### 3.2. Exploratory Data Analysis

The exploratory data analysis based on temporal and spectral features is carried out. The exploratory data analysis is necessary to discover dataset statics, patterns, and all features correlation. The chart and heatmap-based graphs are drawn to explore the dataset.

**Figure 2:**
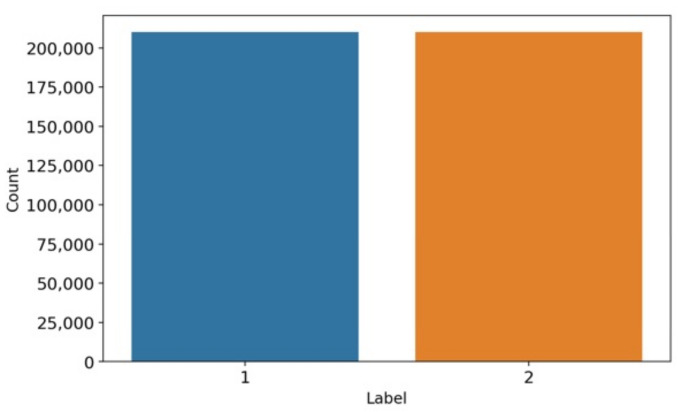
The target label-based data balancing analysis.

The bar chart-based data analysis is visualized in Figure 2. The study shows that target label 1 represents the COPD patient, and target label 2 represents healthy persons. The analysis describes that each class contains a nearly equal number of data distributions and there is no class imbalance problem. The dataset includes 210,000 records for COPD patients and 210,000 records for healthy persons. The equivalent data distribution results in achieving high-performance accuracy scores.

**Figure 3:**
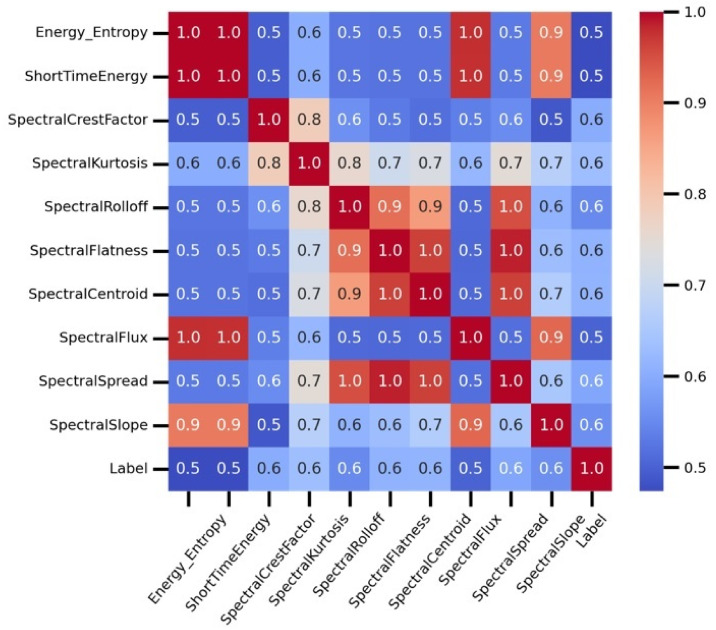
The correlation analysis of temporal and spectral features.

The correlation analysis of temporal and spectral features are demonstrated in Figure 3. The correlation analysis is conducted to determine the association of dataset features that can later cause significant damage during the model fitting. The study determines the strength of a relationship between the temporal and spectral features and computes their correlation association values. Based on the analysis, the features including ’time Rms’, ’gender’, and ’spectral skewness’ are dropped from the dataset due to their low correlation values. The study demonstrates that all used features have a positive correlation score. The features ’energy entropy’, ’short time energy’, ’spectral flux’, and ’spectral slope’ have high positive correlation values, resulting in increased performance for COPD detection.

The temporal and spectral features-based dataset is divided into two portions with a ratio of 0.8 to 0.2. The 80% features data are used to train the machine learning and deep learning methods. The successfully trained applied methods are then tested with the 20% portion of the dataset. The dataset splitting helps overcome the model overfitting issue, resulting in the formation of generalized learning models.

### 3.3. Machine Learning and Deep Learning Models

This study uses DT, LR, GNB, and SVM as machine learning models. The performance of these models is optimized using hyperparameter fine-tuning. In addition, LSTM and GRU deep learning models are also used for experiments. The architecture of deep learning models is also optimized regarding the number of layers, number of neurons, and other parameters.

#### 3.3.1. Decision Tree

DT is a supervised non-parametric technique primarily utilized to solve regression and classification problems. The DT method is based on a tree-like structure that contains branches, connections, and leaf nodes. The DT technique learns decision rules inferred from the dataset. The dataset attributes are mapped onto tree nodes. The tree leaf nodes are based on the target class. The DT method is considered the white box method and has a flow chart-like structure.

#### 3.3.2. Logistic Regression

LR is a statistical machine learning method mostly used to predict binary outcomes. The LR method predicts a dependent data variable by determining the relationship between the independent variables. The LR model is based on the concept of probability of a certain class. The model creates a probability of a certain event occurring given the set of inputs. In LR, the probability is represented by the logistic function. The probabilistic values using the logistic function lie between 0 and 1. Once the model is trained, it can predict the probability of the event occurring for new data.

#### 3.3.3. Gaussian Naive Bayes

The GNB model is a probabilistic model often used for classification and pattern recognition. The GNB is based on the Bayes theorem and assumes that the features of a dataset are independent. The GNB method follows the Gaussian distribution. The Gaussian distribution allows for fast computation and prediction for large datasets. GNB has been used in various applications such as text classification, image classification, and spam filtering. The GNB can achieve high accuracy when the data are not highly complex.

#### 3.3.4. Support Vector Machine

SVM [25] is a supervised learning method that can be used for classification and regression tasks. The SVM method aims to find the best boundary, known as the hyperplane, that separates the data into different classes. The SVM algorithm determines the best boundary by maximizing the margin. The distance between the boundary and the closest data points from each class is known as the support vectors. The main disadvantage of SVM is that it can be sensitive to the choice of kernel function and the value of the regularization parameter.

#### 3.3.5. Long Short-Term Memory

LSTM is an enhanced version of the recurrent neural network [26] and is designed to handle sequential data. The LSTM technique overcomes the problem of vanishing gradients in traditional recurrent neural networks. The LSTM architecture consists of a memory cell, an input gate, an output gate, and a forget gate. These gates allow the LSTM to selectively store, update and retrieve information from the memory cell over a prolonged period of time, allowing it to maintain a long-term context. The limitation of the LSTM method is that it requires a large amount of data to train and can be computationally expensive.

#### 3.3.6. Gated Recurrent Unit

GRU is another recurrent neural network architecture that is designed to address the vanishing gradient problem that plagues traditional recurrent neural networks by introducing gating mechanisms that control the flow of information through the network. The GRU model consists of two gates, the update gate and the reset gate. The update gate controls the proportion of the previous hidden state passed on to the current hidden state. In contrast, the reset gate controls the proportion of the previous hidden state that is reset and replaced with new information. This allows the GRU to selectively retain or discard information from the previous hidden state, making it more efficient and effective at processing sequential data.

The hyperparameter tuning is carried out for both machine learning and deep learning models. Hyperparameter optimization is a recursive process implemented during the training of learning models to optimize their performance for better prediction accuracy. The best-fit hyperparameters of the models used in this study are described in Table 2.

## 4. Results and Discussions

The study experiment results, discussions, and validations of applied machine learning and deep learning models are comparatively analyzed in this section. Many performance metrics are used to evaluate the applied models. The comparative results of machine learning and deep learning models are also analyzed. The performance validation of the outperformed technique with other studies is also conducted.

### 4.1. Experimental Setup

The experiments are conducted in the Google research Colab environment. The used environment is based on a graphical processing unit (GPU) backend with 13 GB RAM, 90 GB disk space, and Intel(R) Xeon(R) system. The Python 3 programming language is utilized to build machine and deep learning models. During the experimental evaluations, the runtime computation, accuracy, precision, recall, and F1 score parameters are measured.

### 4.2. Performance of Employed Models

The experimental results of the machine and deep learning models regarding the accuracy, precision, recall, and F1 scores are presented in Table 3. The analysis demonstrates that the applied GNB technique achieved poor accuracy performance scores in comparison to other models. The GNB technique achieves a 93% score for precision, recall, and F1 metrics, which is also low in comparison. The machine learning-based LR and SVM techniques achieved 89.13% and 89.27%. The deep leaning-based LSTM and GRU also perform well in comparison; however, their performance is lower than the performance of DT. The analysis concludes that the machine learning-based DT technique outperforms other employed models with the highest accuracy of 100% for detecting COPD patients in real-time.

### 4.3. Analysis of Computational Complexity

The comparative analysis of runtime computations of machine learning and deep learning techniques during training is analyzed in Table 4. The study shows that the deep learning LSTM and GRU models require high running time and are thus computationally expensive. LSTM and GRU have the highest time of 988.75 s and 744.23 s, respectively. Comparatively, machine learning models require less training time. The lowest training time is required from the GNB model; however, it also has the lowest accuracy of 82.61%. On the other hand, the training time of the DT classifier is higher than the GNB, but it offers the best prediction accuracy.

### 4.4. K-Fold Cross-Validation Analysis

The k-fold cross-validation technique is applied to validate the performance of applied machine learning and deep learning models, as shown in Table 5. The machine learning-based methods are validated with 10-fold of data. However, the deep learning models are validated with 5-fold data due to the high computational cost. The results analysis demonstrates that machine learning-based LR, GNB, and SVM techniques achieve poor performance during the cross-validations analysis. The validation results of deep learning models are better than LR, GNB, and SVM. The analysis concludes that the DT approach outperforms with a high accuracy score of 99.17 and with the lowest standard deviation of 0.0002 for COPD detection.

### 4.5. Comparison with State-of-the-Art Studies

The performance comparisons analysis of the proposed technique with past applied state-of-the-art approaches is analyzed in Table 6. The past applied deep learning-based LSTM technique achieved 93% accuracy on the same dataset. The analysis reveals that the employed and optimized DT model achieves better results than the previously obtained results for COPD detection employing the UWB data.

## 5. Conclusions

COPD is a life-threatening disease and requires automated detection methods for early diagnosis with high accuracy. This study presents the detection of COPD patients using machine learning and deep learning techniques. The newly created radar-based UWB, collected non-invasively from a distance of 1.5 m, is used in this study. Temporal and spectral features from the data are utilized in this regard to obtain better performance. The exploratory data analysis is applied to discover dataset patterns and features correlation. Experimental results employing DT, LR, GNB, SVM, LSTM, and GRU indicate that the performance of deep learning models is better. However, the best results are obtained using an optimized DT model. The performance of the models is validated using k-fold cross-validation. In addition, a performance comparison with existing methods reveals the better performance of the current approach. In the future, we intend to apply transfer learning- and ensemble learning-based advanced techniques to detect COPD patients.

## Figures and Tables

**Table 1:** The temporal and spectral features-based descriptive dataset analysis.

Feature	Description
Energy Entropy	Energy entropy is the average amount of data acquired in each communication.
Short Time Energy	Energy over a short time period is calculated due to the difficult determination of the energy of a signal since it fluctuates with time.
Time Zero Crossing	Rate The zero-crossing rate refers to the number of times the signal level crosses 0 in a certain amount of time.
Spectral Crest Factor	The tonality of a signal is determined by using the spectral crest factor.
Time Rms	Time Rms are the time-varying sinusoidal complex waveforms where the amplitude changes over time.
Spectral Kurtosis	The frequency domain positions of a sequence of transients are detected using spectral kurtosis.
Spectral Rolloff	The spectral roll-off is used to characterize an energy and frequency link.
Spectral Skewness	The spectrum skewness measures the average number of high frequencies in a signal.
Spectral Flatness	The flatness of a spectrum is measured by using spectral flatness.
Spectral Decrease	The spectral decrease describes the average spectral slope of the rate-map representation.
Spectral Centroid	The spectral centroid is used to quantify a spectrum, which accurately predicts the signal’s brightness.
Spectral Spread	The spread spectrum fills the available frequency range by spreading the provided signal.
Spectral Slope	The signal quality indicator is referred to as spectral slope.
Spectral Flux	The spectral components of the signal are calculated by using the spectral flux.
Label	The target label represents COPD and Health patients.

**Table 2:** Fine-tuned hyperparameters for applied machine learning and deep learning models.

Technique	Hyperparameters	Description
DT	max_depth = 300	The depth level of tree nodes.
	criterion = ‘entropy’	Features splitting method in the tree.
LR	solver = ‘lbfgs’	The algorithm used in the optimization problem.
	max_iter = 100	The number of iterations.
	multi_class = ‘auto’	Target label classification.
GNB	var_smoothing = 1 × 10${}^{-9}$	smoothing that helps tackle the problem of zero probability.
SVM	max_iter = 100	The number of iterations.
	penalty = ‘l2’	Specifies the norm used in the penalization
	loss = ‘squared_hinge’	Specifies the loss function.
LSTM	loss = ‘binary_crossentropy’	Specifies the loss function.
	optimizer = ‘adam’	Loss optimizer function.
	activation = ‘sigmoid’	Output layer activation function
GRU	loss = ‘binary_crossentropy’	Specifies the loss function.
	optimizer = ‘adam’	Loss optimizer function.
	activation = ‘sigmoid’	Output layer activation function

**Table 3:** The performance metrics scores analysis of applied machine and deep learning methods using the unseen test data in real-time.

Model	Accuracy	Precision	Recall	F1 Score
DT	100	99.91	99.89	99.93
LR	89.13	90.11	90.31	90.38
GNB	82.61	83.85	83.91	83.88
SVM	89.27	89.09	89.18	89.20
LSTM	97.01	97.17	97.22	97.12
GRU	99.03	99.07	99.02	99.10

**Table 4:** The runtime computations cost analysis of applied machine and deep learning methods.

Model	Running Time (s)
DT	11.13
LR	16.62
GNB	0.17
SVM	27.20
LSTM	988.75
GRU	744.23

**Table 5:** K-fold cross-validation analysis of the applied machine and deep learning methods.

Model	K Fold	Accuracy	Standard Deviation
DT	10	99.17	0.0002
LR	10	89.31	0.0013
GNB	10	82.86	0.0011
SVM	10	89.60	0.0009
LSTM	5	99.08	0.0053
GRU	5	98.73	0.0073

**Table 6:** The comparative performance results analysis of the proposed technique with state-of-the-art studies.

Reference	Year	Technique	Accuracy
[12]	2022	LSTM	93
Proposed	2023	Decision Tree	100

## Data Availability

Not applicable.
